# MicroRNA MTCO3P38 Inhibits the TMOD1/MMP13 Pathway to Alleviate the Progression of Hepatocellular Carcinoma

**DOI:** 10.7150/jca.100556

**Published:** 2025-01-01

**Authors:** Ping Wu, Guoyuan Li, Deliang Guo, Tao Guo, Chengwei Chai

**Affiliations:** 1 Department of Pediatric Surgery, Guangzhou Women and Children's Medical Center, Guangzhou Medical University, Guangzhou, 510623, Guangdong, China.; 2 Department of Hepatopancreatobiliary Surgery, Zhongnan Hospital of Wuhan University, Wuhan, 430071, Hubei, China.; 3 Department of Pathophysiology, School of Basic Medical Sciences, Shandong Second Medical University, Weifang, 261053, Shandong, China.; 4 Department of Pediatric General Surgery, Guangdong Women and Children Hospital, Guangzhou, 511442, Guangdong, China.

**Keywords:** MTCO3P38, microRNA, TMOD1, MMP13, hepatocellular carcinoma

## Abstract

**Background:** Hepatocellular carcinoma (HCC) is one of the deadliest types of tumors. MicroRNA (miRNA) MTCO3P38 is a novel miRNA derived from the pseudogene MTCO3P38 with 18 nucleotides in length.

**Methods:** The target genes of miR-MTCO3P38 were predicted by Targetscan, RNAhybrid and PITA. Transwell assays verified the effects of miR-MTCO3P38 and its target gene on the migration and invasion of HCC cells. *In vivo*, tumor growth was measured, and the impacts of miR-MTCO3P38 and its target gene on tumor pathology, proliferation, TMOD1/MMP13 pathway and tumor invasion-related factors were evaluated by hematoxylin-eosin staining, immunohistochemistry (Ki67) and western blot.

**Results:** *In vitro*, miR-MTCO3P38 could inhibit the migration and invasion of HCC cells. Mechanistically, miR-MTCO3P38 suppressed the expression of its target gene, tropomodulin 1 (TMOD1), by directly binding the 3'-untranslated region of TMOD1 and then inhibiting the MMP13 pathway. Tumor xenograft model mice were conducted, and *in vivo* assays further confirmed that miR-MTCO3P38 restrained tumor growth and proliferation in HCC by inhibiting the TMOD1/MMP13 pathway.

**Conclusion:** MiR-MTCO3P38 suppresses the TMOD1/MMP13 pathway to alleviate HCC progression.

## Introduction

Hepatocellular carcinoma (HCC) is a malignant tumor with the fourth highest incidence rate and the third highest mortality rate among all tumor sites in China 1. HCC has several well-documented risk factors, including alcohol consumption, adiposis hepatica, dietary exposure to aflatoxin, HBV infection, uncontrolled diabetes, and nonalcoholic fatty liver disease 2. Current therapeutic tools include radiofrequency ablation, sorafenib, surgical resection, liver transplantation and molecular therapy 3. For patients with advanced disease, direct resection or liver transplantation is a commonly used form of treatment. For patients with extrahepatic or vascular invasion, sorafenib offers an additional treatment option, but the toxicity of sorafenib remains an issue 4. The development of HCC is a complex process, so the most appropriate treatment option depends on the disease progression of HCC patients. Excavating more therapeutic and diagnostic markers may be valuable in the treatment of HCC and in improving prognosis.

MicroRNA (miRNA) is a single-stranded noncoding RNA, which is approximately 18-22 nucleotides (nt) in length 5. In the cell nucleus, pri-miRNA that is transcribed from the host miRNA gene transforms to pre-miRNA after being cleaved by the endonuclease Drosha 6. Next, the pre-miRNA is transported to the cytoplasm by the nuclear transporter exportin 5 and is sheared into mature miRNA by endonuclease Dicer 7. There is increasing evidence that microRNAs are important regulators that participate in various cellular and biological processes and are closely related to the pathophysiological processes, diagnosis, and prognosis of liver cancer 8. MicroRNA-26a can serve as a tumor suppressor by downregulating the expression of signal transducer and activator of transcription 3 9. Furthermore, miRNAs can suppress tumor immunity through tumor-associated signaling pathways. MiR-25/93 can repress cGAS expression to mediate hypoxia-induced immunosuppression 10. Several miRNAs, such as miR-30a-30p, miR-342-3p, and miR-15a-3p, were reported to suppress the malignant progression of HCC 11-13. Research on miRNAs that are associated with the diagnosis, prognosis and progression of HCC has obvious clinical significance.

However, a complete picture of the miRNA targetome has not been generated, which defines the number of oncogenes or tumor suppressors targeted by a particular miRNA. To investigate novel miRNAs differentially expressed in HCC, we performed small RNA deep-sequencing analysis in 15 liver tissue samples and a novel miRNA (miR-MTCO3P38) was validated, which was derived from the pseudogene MTCO3P38 and had never been previously annotated in miRbase, and we have reported that miR-MTCO3P38 inhibits HCC malignant progression via STAT3/PTTG1/MYC axis 14. However, the role of miR-MTCO3P38 in HCC remains to be investigated. Tropomodulin 1 (TMOD1) is a member of tropomodulin family, which plays a crucial role in the regulation of thin filament lengths and the formation of actomyosin crossbridge 15. There is evidence that TMOD1 is a prognostic biomarker in cancers, and its high expression contributes to progression of human oral cancer and breast cancer 16, 17. MiR-17-5p promotes the development of gastric cardia cancer by targeting TMOD1 18. So far, the effect of TMOD1 on HCC is still unknown. In our study, TMOD1 was identified as the target gene of miR-MTCO3P38. Through *in vivo* and *in vitro* experiments, we found that miR-MTCO3P38 could suppress HCC progression by inhibiting the TMOD1/MMP13 pathway, providing a promising therapeutic target for the treatment of HCC.

## Materials and Methods

### Plasmid construction

For the construction of the plasmids P1, P2, P3 and P4, the insert DNA fragments containing the sequence of miR-MTCO3P38 were amplified by polymerase chain reaction (PCR). The templates were obtained from the genomic DNA of the HCCLM9 cell line and were extracted with a Genomic DNA Isolation Kit (Biovision, USA). The plasmid vector was pcDNA3.1(-). The restriction enzyme sites were Xho1 and Nde1. To construct the plasmid pcDNA3.1(-)-miR-MTCO3P38, the insert DNA fragments were the quantitative real-time PCR (RT-qPCR) products of miR-MTCO3P38. The restriction enzyme sites that were used were Xho1 and Nde1. This plasmid was verified with DNA sequencing to confirm the RT-qPCR product sequence of miR-MTCO3P38. For the construction of the plasmids pMir-TMOD1-3'-UTR and pMir-TMOD1-3'-UTR-mut, the human TMOD1 3'-UTR (491 bp) with one predicted miR-MTCO3P38 target site was cloned into pMir-Report™ (Applied Biosystems). The mutant 3'-UTR fragments were amplified by using mutated primers. Mlu1 and Hind3 were used as the restriction enzyme sites. All constructs were confirmed by DNA sequencing. The primers for PCRs are presented in Supplementary Table 1.

### Cell culture and transfection

The human HCC cell lines Huh7 and HCCLM9 were obtained from the Cell Bank of Type Culture Collection (CBTCC, Chinese Academy of Sciences, Shanghai, China) and the School of Basic Medical Science of Wuhan University. These cell lines were further characterized by cell viability determination, isozyme detection, mycoplasma testing, and DNA fingerprinting by third-party biology services (GeneCreate Biological Engineering Co., Ltd, Wuhan, China). The cells were cultured with 15% fetal bovine serum (Gibco, USA) and DMEM (HyClone, Utah, USA) and were incubated at 37°C with 5% CO_2_. MiR-MTCO3P38 mimics were designed and synthesized by Viewsolid Biotech (Beijing, China) and were based on the sequence of miR-MTCO3P38. Plasmid and mimic transfection were completed using Lipofectamine 3000 reagent (Invitrogen, Carlsbad, USA). The culture medium was replaced 16-24 h after transfection. The next experiment was performed after two days of cultivation.

### Establishment of HCC mouse model

Twenty-four female BALB/c nude mice (6-8 weeks old, 18-20 g, SPF Biotechnology Co., Ltd, Beijing, China) were randomly divided into LV-NC + miR-NC, LV-NC + miR-MTCO3P38, LV-TMOD1 + miR-NC and LV-TMOD1 + miR-MTCO3P38 group (n=6 in each group). The transfected human Huh7 cells (0.1 mL, 2 × 10^7^ cells/mL) were injected subcutaneously into right flank of nude mice in the corresponding groups. Four weeks later, all mice were anesthetized by isoflurane inhalation and sacrificed by cervical dislocation to obtain tumors, and the tumor weight of the mice was weighed. The length (a) and width (b) of the tumor were measured by vernier caliper (Volume = 1/2 × a × b^2^). All animal experiments were approved by Experimental Animal Welfare and Ethics Committee of Guangzhou Women and Children's Medical Center (No. KTDW-2023-01423).

### Hematoxylin eosin staining (HE staining)

Histopathological changes of tumors were observed by HE staining. Fresh tumor tissues were fixed with paraformaldehyde (4%), embedded in paraffin, and sectioned. Dried slices were immersed in xylene and gradient ethanol solutions. Afterwards, hematoxylin and eosin (Sigma, USA) were used to stain the slices. Then they were dehydrated using an ethanol gradient. At last, the slices were sealed with neutral balsam and observed under an inverted biological microscope (Olympus, Tokyo, Japan).

### Immunohistochemistry (IHC)

The paraffin-embedded sections were dewaxed, and then sodium citrate was employed for antigen repair. Endogenous peroxidase activity was blocked with 3% hydrogen peroxide, and then sections were cut with 3% bovine serum albumin (BSA) to reduce non-specific staining. Subsequently, sections were incubated with Ki67 primary antibody (ab16667, Abcam, Cambridge, MA, USA) at 4°C overnight. Afterwards, sections were incubated with goat anti-rabbit IgG secondary antibody (Abcam) at room temperature, and then the chromogenic solution DAB was added. Afterwards, the sections were re-stained with hematoxylin, dehydrated and sealed with neutral gum. Staining results were observed under an optical microscope (Olympus).

### RT-qPCR

Total RNA was extracted with TRIzol reagent (Invitrogen, Carlsbad, USA) from human liver tissues and cells. The reverse transcription (RT) reaction of miR-MTCO3P38 was completed by using the miRNA cDNA Synthesis Kit with Poly(A) Polymerase Tailing (Abmgood, Canada). To construct the plasmid pcDNA3.1(-)-miR-MTCO3P38, the downstream reverse transcription reaction primer was redesigned because the primer sequence in the miRNA cDNA Synthesis Kit was not provided by the manufacturer. RT-qPCR was performed with SYBR Select Master Mix (Life Technologies, USA). Internal controls for the TMOD1 mRNA and miR-MTCO3P38 were GAPDH and RNU6B, respectively. The primers for RT and RT-qPCR reactions are listed in Supplementary Table 1.

### Western blot and northern blot

For western blot analysis, total protein was extracted with RIPA and 1% PMSF from transfected cells or tissues. Protein concentration was measured by a bicinchoninic acid (BCA) protein kit (Thermo Scientific, USA). SDS-PAGE and PVDF membranes (Millipore, Burlington, USA) were used for protein separation. PVDF membranes were blocked with 5% skimmed milk at 25℃ for 1 h, and then incubated with the primary antibodies (Abcam) at 4℃ overnight. The antibodies are presented in Supplementary Table 2. Afterwards, the membranes were washed and incubated with goat anti-rabbit IgG secondary antibody (Abcam) at 25℃ for 1 h. Finally, membranes were added with ECL for developing, and then exposed to capture pictures.

For northern blot analysis, total RNA was extracted from HCCLM9 cells transfected with the plasmids P1, P2, P3, P4 and pcDNA3.1(-). Amersham Hybond-N+ membranes (GE, Boston, USA) were used for the transfer. The membranes underwent detection using a DIG Northern Starter Kit (Roche, FD2001, Shanghai, China) following the manufacturer's protocol. The sequences of the hybridization probe are as follows: miR-MTCO3P38: 5'-GCCCUCUCAGCCCUCCUA-Digoxin-3'; and U6: 5'-AUAUGGAACGCUUCACGAAUU-Digoxin-3'.

### Luciferase activity assay

A recombinant pMIR-Report luciferase, pMir-TMOD1-3'-UTR or pMir-TMOD1-3'-UTR-mut plasmid and miR-MTCO3P38 or miR-NC were cotransfected into Huh7 and HCCLM9 cells for 48 h. The cells were lysed and subjected to luciferase activity assays by using a Dual-Glo system (Promega, WI, USA) according to the manufacturer's instructions.

### Cell invasion and migration assay

For cell invasion assays, transwell chambers were precoated with 200 μg/mL Matrigel (BD Biosciences, USA) and incubated overnight. Huh7 and HCCLM9 cells were cultured in serum-free medium in the upper chambers, and 500 μL of DMEM containing 10% fetal bovine serum was added to the lower chamber. After incubation for 24 h, the cells were fixed with 4% paraformaldehyde and stained with 0.1% crystal violet. Phase-contrast microscopy was used for counting the stained cells from three different visual fields of each filter. For cell migration assays, cells were cultured in 6-well plates. Confluent cell monolayers were disrupted by standardized wound scratching using a sterile 10 μL pipette tip and cultured with serum-free medium. The area of the wound was measured at 0 h and 24 h.

### Statistics

Statistical analyses were completed with GraphPad Prism 5 software. Data are presented as the mean ± standard deviation (S.D.) from at least three independent experiments. A paired t-test was performed to calculate the expression differences between two groups. One-way ANOVA and Tukey's test were carried out for comparisons between multiple groups.

## Results

### MiR-MTCO3P38 overexpression inhibits cell invasion and migration through a novel pathway involving MMP13 in HCC

**Figure 1:**
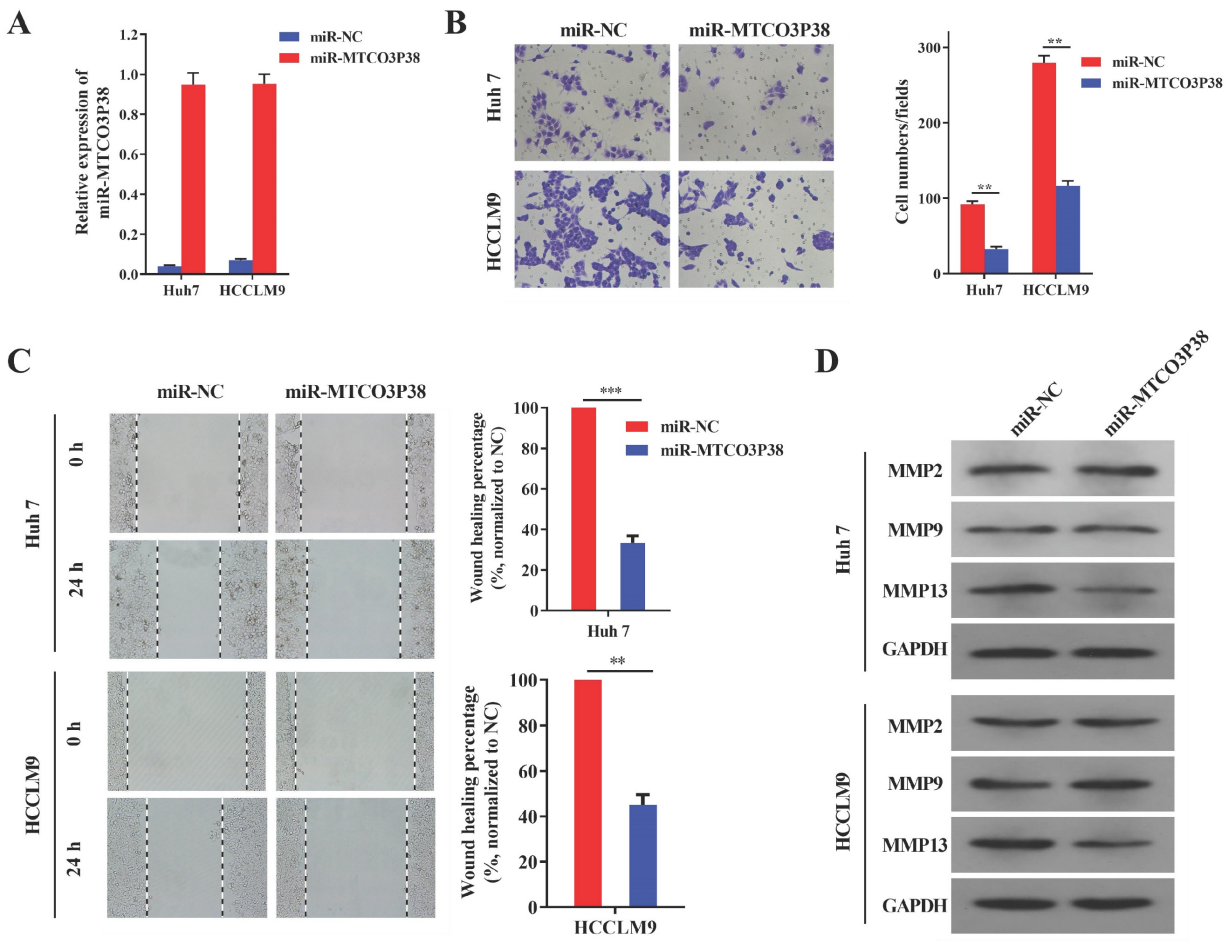
** MiR-MTCO3P38 represses hepatoma cell invasion and migration *in vitro*. (A)** The relative expression of miR-MTCO3P38 in HCC cells transfected with the negative control and miR-MTCO3P38 mimics for 48 h.** (B)** Cell invasion assays with HCC cells transfected with miR-MTCO3P38 mimics for 48 h. ^**^p < 0.01 ^***^p < 0.001 **(C)** Representative images of wound healing assays with HCC cells after transfection with miR-MTCO3P38 mimics for 24 h. ^**^p < 0.01 ^***^p < 0.001 **(D)** MMP-2, MMP-9 and MMP-13 protein expression levels in Huh7 cells and HCCLM9 cells transfected with miR-MTCO3P38 mimics for 48 h.

MiR-MTCO3P38 mimics were synthesized to study the function of miR-MTCO3P38 in HCC cell lines. RT-qPCR showed that miR-MTCO3P38 expression was extremely upregulated in Huh7 and HCCLM9 cell lines after transfection with miR-MTCO3P38 mimics (Fig. 1A). Transwell assays were then carried out and showed that miR-MTCO3P38 overexpression dramatically inhibited Huh7 and HCCLM9 cell invasion compared to those of control cells (Fig. 1B). Wound healing assays were performed and showed that miR-MTCO3P38 overexpression dramatically inhibited Huh7 and HCCLM9 cell migration compared to those of control cells (Fig. 1C). Matrix metalloproteinases (MMPs) are a family of enzymes that have been recognized as important factors in the oncogenic and metastatic processes of HCC 19. MMP2, MMP9 and MMP13 proteins play significant roles in liver cancer invasion and migration. To investigate the influence of miR-MTCO3P38 on MMP family proteins, miR-MTCO3P38 was overexpressed in the Huh7 and HCCLM9 cell lines. The results showed that MMP13 was downregulated upon miR-MTCO3P38 overexpression, while the expression of MMP2 and MMP9 showed no change compared to those of control cells (Fig. 1D). These results indicate that miR-MTCO3P38 inhibits cell invasion and migration and that this process involves MMP13.

### TMOD1 is a direct target of miR-MTCO3P38 in HCC cells

**Figure 2:**
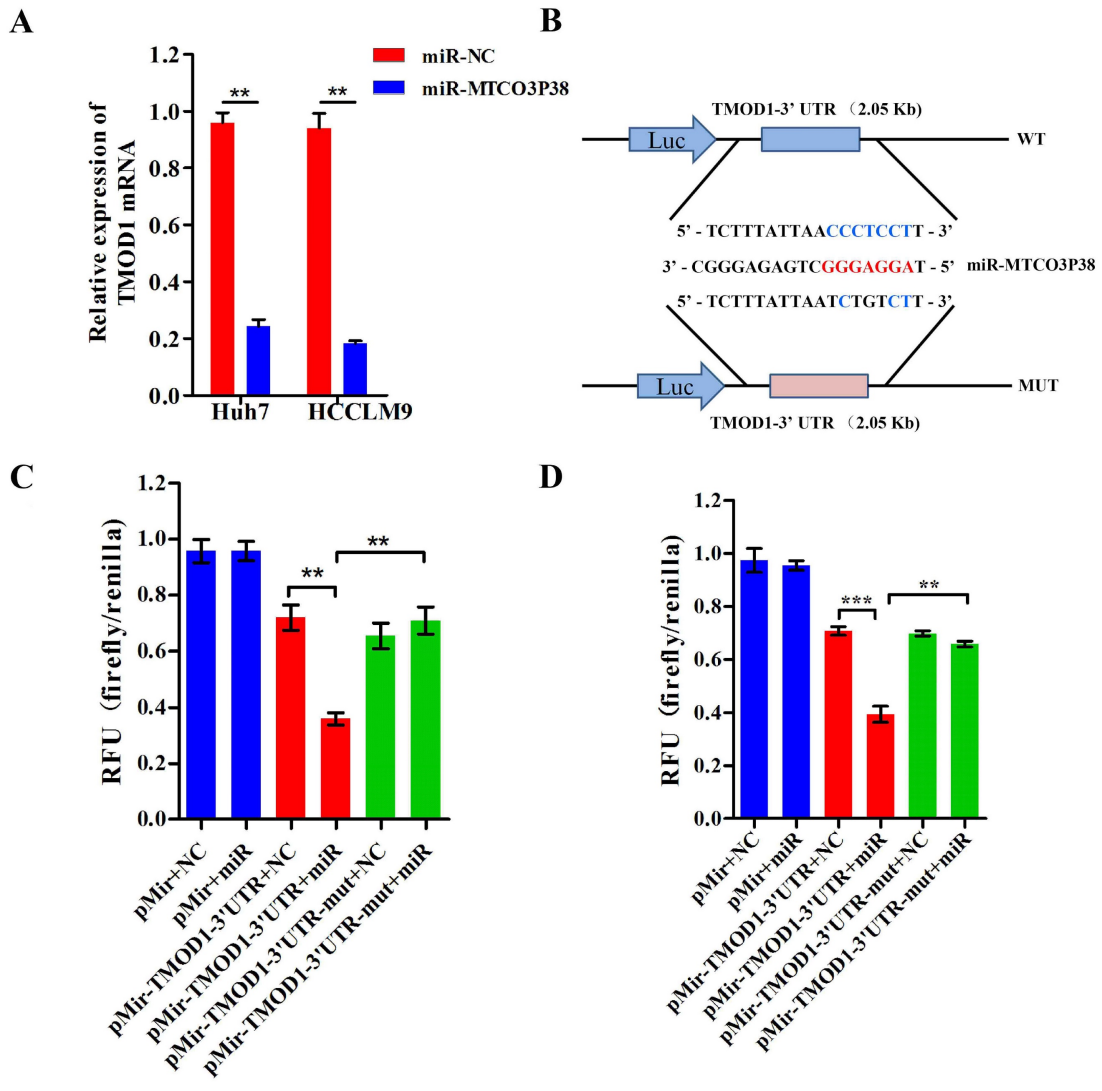
** TMOD1 is a direct target of miR-MTCO3P38 in Huh7 and HCCLM9 cells. (A)** The relative expression of TMOD1 mRNA in HCC cells transfected with the negative control and miR-MTCO3P38 mimics for 48 h. ^**^P < 0.01 **(B)** Wild-type and mutant sequences of the TMOD1 3'-UTR for the luciferase reporter assay. Red bases indicate the seed region of miR-MTCO3P38. **(C)** Luciferase reporter assay analyzing the interaction between miR-MTCO3P38 and the TMOD1 3'-UTR in Huh7 cells. WT: wild-type, MUT: mutant sequences, miR: miR-MTCO3P38 mimics, NC: negative control for mimics, pMir: pMir-Report plasmid. ^**^P < 0.01 **(D)** Luciferase reporter assay analyzing the interaction between miR-MTCO3P38 and the TMOD1 3'-UTR in HCCLM9 cells. WT: wild-type, MUT: mutant sequences, miR: miR-MTCO3P38 mimics, NC: negative control for mimics, pMir: pMir-Report plasmid. ^**^P < 0.01.

To identify genes that are targeted by miR-MTCO3P38, we performed gene expression analysis on Huh7 cells that had or had not been treated with miR-MTCO3P38 mimics. Next, the differentially expressed genes with a fold change > 2 and q-value < 0.05 were selected and analyzed to determine the targeted gene with the miRNA prediction software Targetscan, RNAhybrid and PITA. The mRNAs (NM_003275 and NM_001166116) of TMOD1 were predicted as the targeted genes of miR-MTCO3P38 by Targetscan (91 score), RNAhybrid (molar free energy = -21.4 KJ/mol) and PITA (Supplementary Table 4). In addition, CFH, DNAH12, IGFBP5 and LYPD1 were either downregulated or predicted to be downregulated (Supplementary Table 3 and Supplementary Table 4). To verify the gene expression results, TMOD1 mRNA expression was measured with RT-PCR in the Huh7 and HCCLM9 cell lines. TMOD1 was downregulated with the overexpression of miR-MTCO3P38 in both cell lines compared to that of control cells (Fig. 2A). The putative binding sites of miR-MTCO3P38 in the TMOD1 3'-UTR were identified (Fig. 2B). Then, luciferase assays were performed to verify whether miR-MTCO3P38 could bind to the 3'-UTR of TMOD1, which illustrated that overexpression of miR-MTCO3P38 significantly suppressed the luciferase activity of the wild-type TMOD1 3'-UTR, whereas it had no impact on the luciferase activity of the mutant TMOD1 3'-UTR in Huh7 cell lines (Fig. 2C). Similarly, the same result was also presented in HCCLM9 cells (Fig. 2D). Above results suggest that TMOD1 is a direct target gene of miR-MTCO3P38.

### Alteration of TMOD1 expression reverses the influence of miR-MTCO3P38 on cell invasion and migration in HCC cells

**Figure 3:**
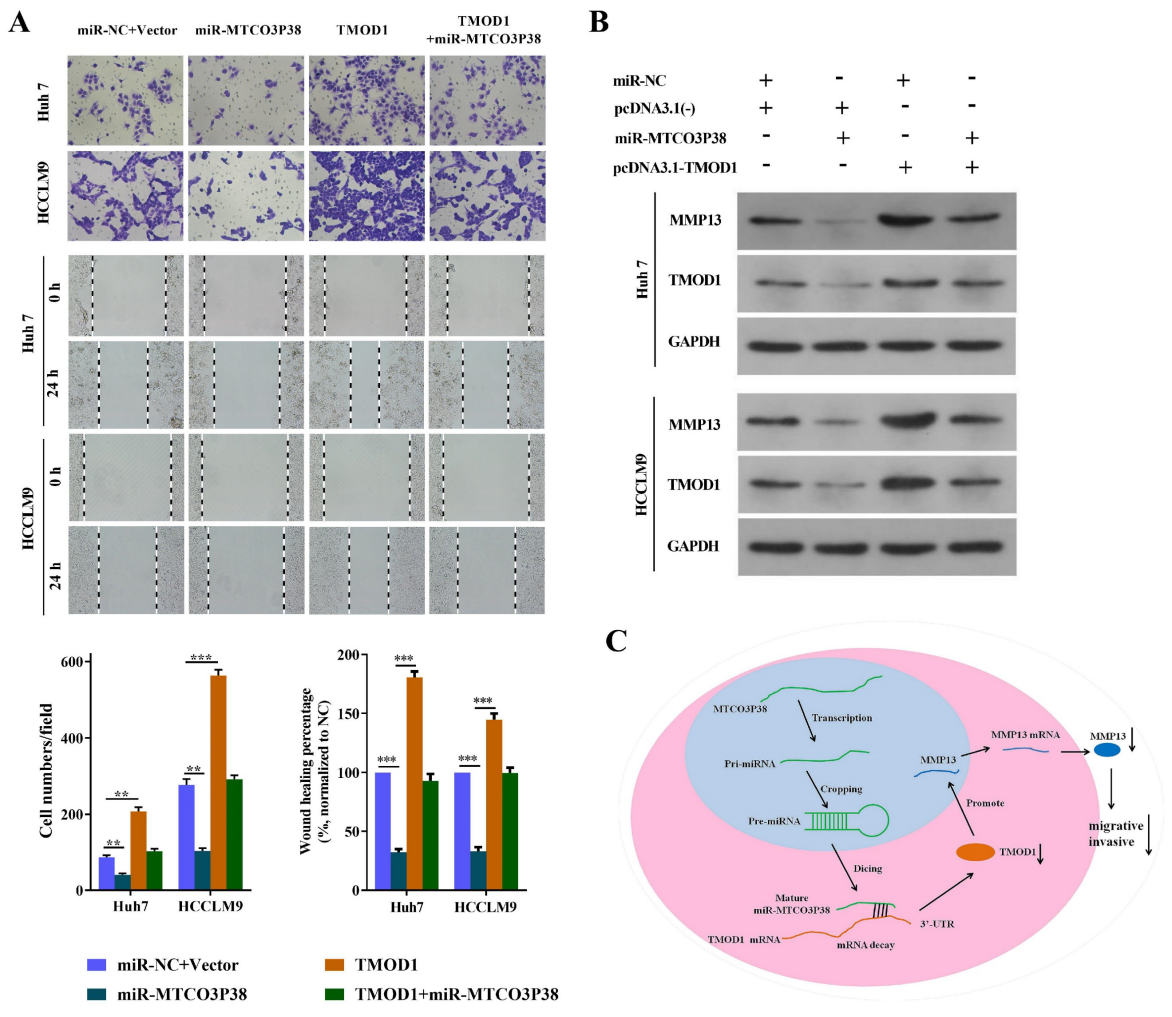
** MiR-MTCO3P38 suppresses migration and invasion of HCC cells by targeting TMOD1. (A)** Representative images of the invasion and migration of Huh7 and HCCLM9 cells (transfected with the following molecules: the negative control; miR-MTCO3P38 mimics; pcDNA3.1(-) - TMOD1; and miR-MTCO3P38 mimics and pcDNA3.1(-) - TMOD1) by wound healing assays. after **(B)** MMP-13 and TMOD1 protein expression levels in transfected Huh7 cells and HCCLM9 cells for 48 h. **(C)** A model depicting how miR-MTCO3P38 is produced and how it decreases MMP13 by targeting TMOD1 mRNA, thus inhibiting the invasion and migration of human HCC cells. ^**^P < 0.01 ^***^P < 0.001 *vs.* miR-NC+Vector.

To further demonstrate that the effect of miR-MTCO3P38 on cell migration and invasion occurred by directly targeting TMOD1, we completed a series of rescue experiments. The Huh7 and HCCLM9 cells were divided into miR-NC + Vector (transfected with the miR-NC + vector), miR-MTCO3P38 (transfected with miR-MTCO3P38 mimics + vector), TMOD1 (transfected with miR-NC + pcDNA3.1(-) - TMOD1), and TMOD1 + miR-MTCO3P38 (transfected with miR-MTCO3P38 mimics + pcDNA3.1(-) - TMOD1). The results indicated that TMOD1 promoted cell invasion and migration, while overexpression of miR-MTCO3P38 further reversed the situation (Fig. 3A). Western blot assays showed that the overexpression of TMOD1 could rescue the downregulation of MMP13, which could be attributed to the increase in miR-MTCO3P38 expression (Fig. 3B). All the results showed that TMOD1 modulation can reverse the function of miR-MTCO3P38, suggesting that the effect of miR-MTCO3P38 on cell migration and invasion occurs through the direct targeting of TMOD1 in HCC. Mechanistically, miR-MTCO3P38 suppressed the expression of its target gene, TMOD1, by directly binding the 3'-untranslated region of TMOD1 and then inhibiting the MMP13 pathway (Fig. 3C).

### MiR-MTCO3P38 inhibits tumor growth and development by suppressing the TMOD1/MMP13 pathway *in vivo*

**Figure 4:**
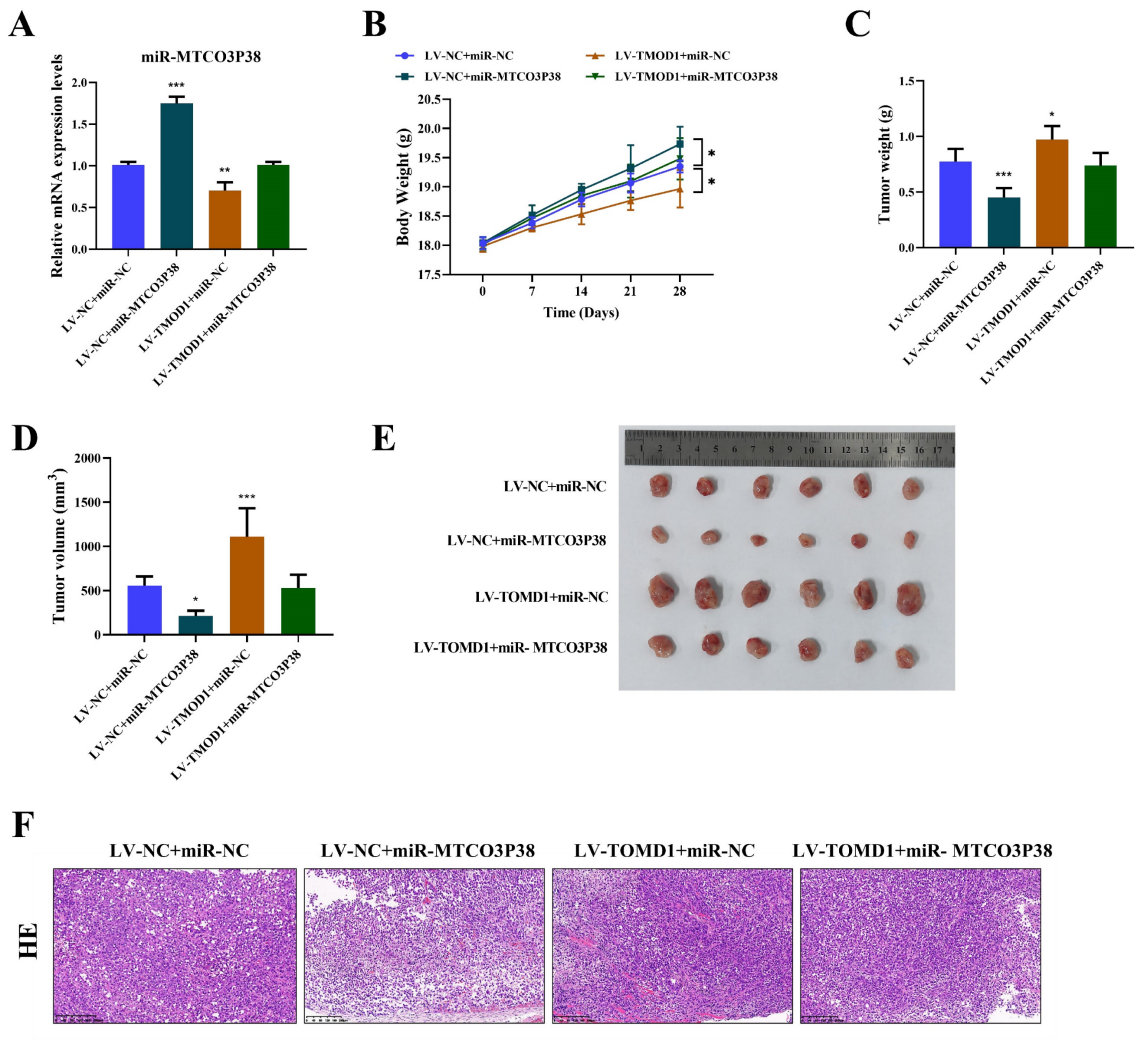
** MiR-MTCO3P38 inhibits tumor growth and development by suppressing TMOD1/MMP13 pathway *in vivo*. (A)** Detection of miR-MTCO3P38 expression by RT-qPCR. **(B)** Body weight of mice. **(C)** Tumor weight in mice. **(D)** Tumor volume in mice. **(E)** Tumor size in mice. **(F)** Representative images of the structure changes of tumor tissues by hematoxylin-eosin staining (magnification: 200×, scale: 200 μm).

Likewise, the inhibitory effect of miR-MTCO3P38 on HCC by directly targeting TMOD1 was further verified by *in vivo* assays. Each group of mice were injected with the corresponding transfected Huh7 cells. It was discovered that the level of miR-MTCO3P38 was significantly increased in HCC mice, and overexpression of TMOD1 led to the down-regulation of miR-MTCO3P38 in HCC (Fig. 4A). Mice weight, the size, weight, and volume of tumors were measured, and the pathological changes of the tumor tissue were observed by HE staining, demonstrating that overexpression of miR-MTCO3P38 inhibited tumor growth and development in HCC mice, which was weakened by TMOD1 overexpression (Fig. 4B-F).

**Figure 5:**
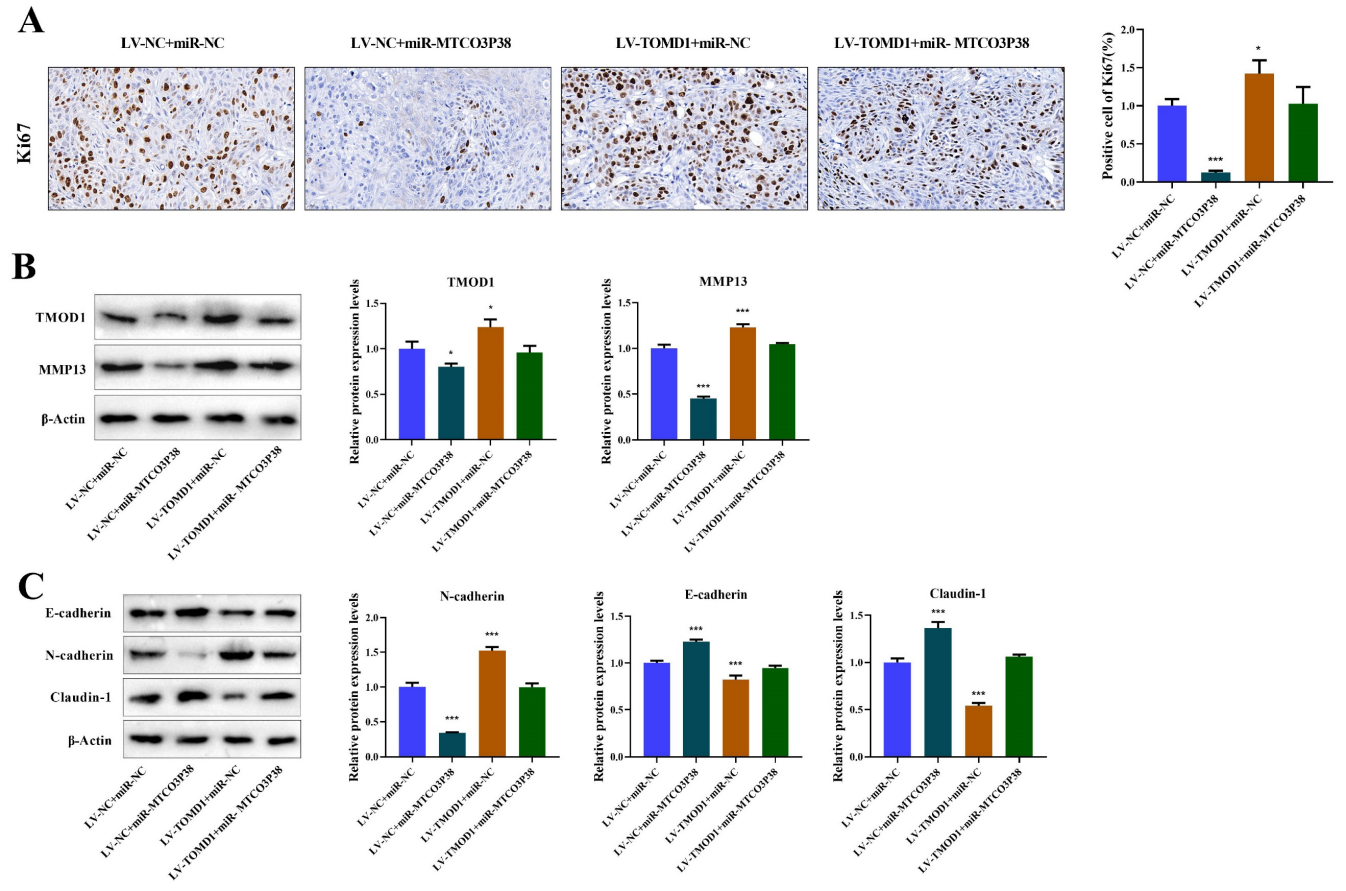
** MiR-MTCO3P38 inhibits tumor growth and development by suppressing TMOD1/MMP13 pathway *in vivo*. (A)** Detection of Ki67 positive cells (brown-stained cells) by immunohistochemistry (magnification: 400×, scale: 20 μm). **(B)** Detection of TMOD1 and MMP13 protein expression by western blot. **(C)** Relative protein expressions of tumor invasion-related proteins (E-cadherin, N-cadherin, and Claudin-1) were tested by western blot. ^*^P < 0.05 ^**^P < 0.01 ^***^P < 0.001 *vs.* LV-NC + miR-NC group.

Additionally, IHC was performed to detect Ki67 positive cells to evaluate tumor proliferation, demonstrating that TMOD1 notably reversed the suppressive impact exerted by miR-MTCO3P38 on tumor proliferation (Fig. 5A). The protein expression of TMOD1/MMP13 pathway and tumor invasion-related factors were detected by western blot, illustrating that overexpression of TMOD1 rescued the downregulation of TMOD1 and MMP13, which was caused by high expression of miR-MTCO3P38 (Fig. 5B). In terms of tumor invasion, overexpression of miR-MTCO3P38 significantly increased E-cadherin and Claudin-1 expression and reduced N-cadherin level, while overexpression of TMOD1 reversed the situation (Fig. 5C). Above results illustrate that miR-MTCO3P38 inhibits HCC progression in mice by down-regulating the TMOD1/MMP13 pathway.

## Discussion

Hepatocellular carcinoma (HCC) is one of the most common malignancies worldwide, with high incidence and poor prognosis 20. The dysregulation of miRNAs in HCC remains complex and urgently needs to be explored 21. The use of small RNA deep-sequencing microarrays to find differentially expressed miRNAs in a variety of tumors is a common method in cancer research, such as colorectal cancer, glioma and HCC 22-24. In the past, noncoding RNAs and pseudogenes were considered as nonfunctional 'junk', but some have been to have molecular biological functions now 25, 26. Increasingly, non-coding RNAs have been found to play a substantial role in the development and progression of cancers 27. Among them, miRNAs are promising options for targeted therapy of cancers 28.

In recent years, plenty of miRNAs have presented their promising clinical therapeutic potential in HCC, and various approaches were carried out to seek novel miRNAs. In terms of diagnosis and prognosis, many miRNAs have significant expression differences in liver cancer tissues, adjacent nontumor tissues and normal tissues, and some of these miRNAs can be used clinically as serum diagnostic markers for HCC. MiR-122, miR-221, miR-199a/b, mi-125a/b, and miR-222 could act as biomarkers in the early stage of HCC diagnosis/prognosis 29. MicroRNA-802 promotes the proliferation of HCC cells and accelerates HCC progression by targeting RUNX3^30^. MiR-766 promotes the progression of HCC by regulating NR3C2 expression, which is related to the prognosis of HCC 31. Most importantly, miRNAs act as nucleic acid-based drugs with great potential for the treatment of HCC. Adipose tissue-derived mesenchymal stem cells transfected with miR-122 can make HCC cells more sensitive to chemotherapy drugs 29, 32. Recently, several preclinical formulations have shown promise with low toxicity profiles and with the ability to deliver their payload to the target site 25, 33. MiR-495 regulates cell cycle and senescence by targeting CTRP3 to inhibit HCC cell growth, and *in vivo* assays illustrate that miR-495 has a better therapeutic effect on HCC in xenograft 34. miR-509-3p inhibits migration and invasion of HCC cell lines (HepG2 and HCCLM3 cells) and suppresses cancer metastasis *in vivo* 35. The microRNA-4651-FOXP4 axis inhibits HCC growth and promotes apoptosis, which may be a potential target for the treatment of HCC 36. Herein, the influence of miR-MTCO3P38 on tumor behaviors was evaluated *in vitro*, demonstrating that miR-MTCO3P38 could inhibit the migration and invasion of HCC cells.

Gene expression profiles and targeted gene predictor software found that there were 5 genes (TMOD1, CFH, DNAH12, IGFBP5 and LYPD1) that may be targeted by miR-MTCO3P38. Among them, TMOD1 was of particular interest, which had a relatively high expression level in liver cells. Thus, TMOD1 was chosen for further study and the results demonstrated that miR-MTCO3P38 downregulated TMOD1 expression by interacting with the 3'-UTR of TMOD1 mRNA. Subsequently, rescue assays proved that miR-MTCO3P38 directly downregulated TMOD1 expression, leading to the repression of the expression of MMP13. It has been reported that TMOD1 can upregulate MMP13 by inhibiting the degradation of β-catenin degradation; the subsequent accumulation of β-catenin in the cell nucleus promotes MMP13 mRNA expression 17. Therefore, it is reasonable to hypothesize that miR-MTCO3P38 inhibits the migration and invasion of HCC cells by targeting the TMOD1 gene, and thus influencing the β-catenin/MMP13 pathway. Afterwards, *in vitro* and *in vivo* experiments showed that overexpression of TMOD1 reversed the inhibitory effects of miR-MTCO3P38 on migration and invasion of HCC cells and tumor growth, illustrating that miR-MTCO3P38 restrained tumor growth and development by targeting TMOD1/MMP13 pathway. Evidences have shown that high expression of TMOD1 is a key prognostic marker for patients with oral squamous cell carcinoma and is closely associated with regional lymph node metastasis 16. In addition, a gene model involving TMOD1 have been identified as favorable diagnostic markers for identifying anaplastic large-cell lymphoma 37. MiR-588 was able to inhibit the proliferation of HCC cells by suppressing the expression of TMOD1 38. Combined with above results, it is concluded that miR-MTCO3P38 can inhibit the TMOD1/MMP13 pathway to alleviate the progression of HCC.

## Conclusion

Herein, TMOD1 was identified as the target gene of miR-MTCO3P38. Through *in vivo* and *in vitro* assays, miR-MTCO3P38 can inhibit HCC progression via the miR-MTCO3P38/TMOD1/MMP13 axis. Our findings open new avenues for future studies of this novel miRNA, offering a potential therapeutic target for HCC.

## Supplementary Material

Supplementary tables.

## Abbreviations

HCC: hepatocellular carcinoma
RT-QPCR: quantitative real-time polymerase chain reaction
MiRNA: microRNA
nt: nucleotides
3'-UTR: 3'-untranslated region
TMOD1: tropomodulin 1
