# Supplementary material for: MicroRNA MTCO3P38 Inhibits the TMOD1/MMP13 Pathway to Alleviate the Progression of Hepatocellular Carcinoma
Source: J Cancer. 2025 Jan 1;16(2):486–95. doi: 10.7150/jca.100556 (PMC11685701; doi:10.7150/jca.100556)
Supplement: Supplementary file 1 — Supplementary tables. [file jcav16p0486s1.zip › Supplementary Table 1.docx]

**Table S1 Primer sequences used in this study**

| **Name** | **Sequences (5'-3')** |
| --- | --- |
| F-miR-MTCO3P38-qPCR | ACACTCCAGCTGGGTAGGAGGGCTGAGA |
| R-miR-MTCO3P38-qPCR | GGCCAACCGCGAGAAGATGTTTTTTTTT |
| F-U6-qPCR | GCTTCGGCAGCACATATACTAAAAT |
| R-U6-qPCR | CGCTTCACGAATTTGCGTGTCAT |
| F-TMOD1-qPCR | ACAGAGGAAGAGCTGAGGACCCTGG |
| R-TMOD1-qPCR | GCTTCTGCTTAGGAACCCAGACCTT |
| F-GAPDH-qPCR | ACGGATTTGGTCGTATTGGGC |
| R-GAPDH-qPCR | CTCGCTCCTGGAAGATGGTGAT |
| F-pcDNA3.1-miR-MTCO3P38 | AATTCTCGAGACACTCCAGCTGGGTAGGAGGGCT |
| R-pcDNA3.1-miR-MTCO3P38 | GGAATTCCATATGGGCCAACCGCGAGAAGATG |
| F-P1-1Kb | TTTTCTCGAGCATGGCAGGGTAGTAGTAGG |
| R-P1-1Kb | CCCCAAGCTTTTAGCCAAATCAACTCTGAA |
| F-P2-500bp | TTTTCTCGAGCCATAGATTCCATCAGAGGT |
| R-P2-500bp | AGGGAAGCTTGGGATTTGTATCCAGAATAA |
| F-P3-250bp | TTTTCTCGAGGAGTTGGGGTTAGACTAGAG |
| R-P3-250bp | AGGAAAGCTTGCAGAAATTGCAACCCTCAT |
| F-P4 | TTTTCTCGAGACAGAGACGTATAAGAATAG |
| R-P4 | AGGAAAGCTTTAATGACCCACCAAACACAT |
| 1F-pMir-TMOD1-WT | CCAAACGCGTAGTCACAGAAGTTGAATCTGGT |
| 2R-pMir-TMOD1-WT | TGTGAAGCTTTTTGTTGCCTGGGATTGGTAA |
| 1R-pMir-TMOD1-MUT | TTCAAAATTCCAAGACAGATTAATAAAGAAT |
| 2F-pMir-TMOD1-MUT | ATTCTTTATTAATCTGTCTTGGAATTTTGAA |
| Northern Blot Probe-miR-MTCO3P38 | 5’-GCCCUCUCAGCCCUCCUA-Digoxin-3’ |
| Northern Blot Probe-U6 | 5’-AUAUGGAACGCUUCACGAAUU-Digoxin-3’ |
| mimics-miR-MTCO3P38-Sense | UAGGAGGGCUGAGAGGGC |
| mimics-miR-MTCO3P38-Antisense | GCCCUCUCAGCCCUCCUA |
| Negative mimics Control-Sense | UUCUCCGAACGUGUCACGUTT |
| Negative mimics Control-Antisense | ACGUGACACGUUCGGAGAATT |
