# Supplementary material for: MicroRNA MTCO3P38 Inhibits the TMOD1/MMP13 Pathway to Alleviate the Progression of Hepatocellular Carcinoma
Source: J Cancer. 2025 Jan 1;16(2):486–95. doi: 10.7150/jca.100556 (PMC11685701; doi:10.7150/jca.100556)
Supplement: Supplementary file 1 — Supplementary tables. [file jcav16p0486s1.zip › Supplementary Table 2.docx]

**Table S2 Primary antibodies for western blot**

| **Name** | **No.** | **Manufacturer** |
| --- | --- | --- |
| GAPDH | ab9485 | Abcam |
| MMP-2 | ab92536 | Abcam |
| MMP-9 | ab76003 | Abcam |
| MMP-13 | ab39012 | Abcam |
| TMOD1 | ab230276 | Abcam |
| E-cadherin | ab40772 | Abcam |
| N-cadherin | ab76011 | Abcam |
| Claudin-1 | ab211737 | Abcam |
| β-Actin | ab8226 | Abcam |
